# Tumour size measurement in a mouse model using high resolution MRI

**DOI:** 10.1186/1471-2342-12-12

**Published:** 2012-05-30

**Authors:** Mikael Montelius, Maria Ljungberg, Michael Horn, Eva Forssell-Aronsson

**Affiliations:** 1 Department of Radiation Physics, Institute of Clinical Sciences, Sahlgrenska Cancer Centre, Sahlgrenska Academy, University of Gothenburg, Sahlgrenska University Hospital, Gothenburg, SE-413 45, Sweden; 2 Division of Medical Physics and Medical Engineering, Sahlgrenska University Hospital, Gothenburg, SE-413 45, Sweden; 3 Department of Radiology, University of Würzburg, Würzburg, Germany

**Keywords:** Animal model, Magnetic resonance, Volume determination, Cancer

## Abstract

**Background:**

Animal models are frequently used to assess new treatment methods in cancer research. MRI offers a non-invasive *in vivo* monitoring of tumour tissue and thus allows longitudinal measurements of treatment effects, without the need for large cohorts of animals. Tumour size is an important biomarker of the disease development, but to our knowledge, MRI based size measurements have not yet been verified for small tumours (10^−2^–10^−1^ g). The aim of this study was to assess the accuracy of MRI based tumour size measurements of small tumours on mice.

**Methods:**

2D and 3D T2-weighted RARE images of tumour bearing mice were acquired *in vivo* using a 7 T dedicated animal MR system. For the 3D images the acquired image resolution was varied. The images were exported to a PC workstation where the tumour mass was determined assuming a density of 1 g/cm^3^, using an in-house developed tool for segmentation and delineation. The resulting data were compared to the weight of the resected tumours after sacrifice of the animal using regression analysis.

**Results:**

Strong correlations were demonstrated between MRI- and necropsy determined masses. In general, 3D acquisition was not a prerequisite for high accuracy. However, it was slightly more accurate than 2D when small (<0.2 g) tumours were assessed for inter- and intraobserver variation. In 3D images, the voxel sizes could be increased from 160^3^ μm^3^ to 240^3^ μm^3^ without affecting the results significantly, thus reducing acquisition time substantially.

**Conclusions:**

2D MRI was sufficient for accurate tumour size measurement, except for small tumours (<0.2 g) where 3D acquisition was necessary to reduce interobserver variation. Acquisition times between 15 and 50 minutes, depending on tumour size, were sufficient for accurate tumour volume measurement. Hence, it is possible to include further MR investigations of the tumour, such as tissue perfusion, diffusion or metabolic composition in the same MR session.

## Background

The efficacy of new tumour treatment methods are usually tested in animal models prior to clinical trials(e.g. [1-3]). The end-point studied is often the change in tumour size using mice with subcutaneous xenografts of human tumour tissue (e.g. [4]). Two or three perpendicular diameters of the tumour are measured and volume is calculated under assumptions of e.g. ellipsoidal tumour shape (e.g. [5]). These methods are fast, inexpensive, and do not require anaesthesia. Studies on orthotopic and metastatic tumour models have increased [6-9]. Generally, such studies exclude external volume measurements and require non-invasive imaging techniques, such as magnetic resonance imaging (MRI), computed tomography (CT), positron emission tomography or single photon emission computed tomography[10,11].

MicroCT imaging allows accurate measurement of very small tumour volumes (~10^−2^ g) in short acquisition time [12], but the radiation exposure might affect tumour growth and thus confound the interpretation of the treatment results. MRI does not expose the animal to ionizing radiation and gives no known side effects which opens the possibility to follow the same animal by consecutive non-invasive measurements in longitudinal studies. In addition, MRI has intrinsic contrast abilities which can be used to assess tumour tissue characteristics, e.g. diffusion, perfusion and metabolic properties, parameters reflecting cell proliferation, apoptosis, vascularisation, etc. [11, 13-21].

Multiparametric MR imaging requires optimised MR methods for minimum time consumption. Small tumours require careful attention regarding imaging parameters due to the partial volume effect (PVE). To our knowledge, there is a lack of information on the accuracy and precision of MRI based size measurements on small tumours.

The aim of this study was 1) to develop and assess the accuracy of an MRI based method for *in vivo* measurement of tumour size in a small animal tumour model, and 2) to investigate the possibility to reduce acquisition time by reducing image resolution, without affecting the accuracy.

## Methods

### Tumour models and experimental setup

The study was performed on 17 tumour bearing female, athymic BALB/c and SCID mice s.c. inoculated in the neck/back region with the human midgut carcinoid cell line, GOT1 (n = 15), or the medullary thyroid carcinoma, GOT2 (n = 2) under anaesthesia using tribromoethanol (Avertin®, Winthrop Laboratories, Surbiton, UK). Animals had free access to water and standard diet.

Within two days after MR imaging, animals were sacrificed by surgical incision of the heart after i.p. injection of sodium pentobarbital (Apoteket Farmaci, Stockholm, Sweden). Gauge block measurements of the length, *l*, width, *w* and height, *h* of the tumours were performed. Assuming ellipsoidal tumours, the volume *V = (π/6)·l·w·h*, was determined and converted to mass, *m*_*GB*_, assuming a tumour density of 1.0 g/cm^3^. The tumour was then surgically removed and weighed for reference tumour mass, *m*_*T*_, determination. All procedures were approved by the local Animal ethics committee in Gothenburg.

### MR imaging

MRI was performed using a 7 T MR system (Bruker BioSpin MRI GmbH, Ettlingen, Germany; software: ParaVision 5.0) equipped with water cooled gradient coils (maximum gradient strength 400 mT/m). A 72 mm volume coil was used for transmission and an actively decoupled 4 channel array rat brain coil was used for signal receiving (RAPID Biomedical GmbH, Rimpar, Germany). Proper animal positioning was assured using a fast gradient-echo localizer scan. All other imaging experiments were based on a respiration triggered and fat suppressed T2-weighted rapid acquisition with relaxation enhancement (RARE) sequence [22]. A 2D set of axial images was acquired (TR = 3438–4186 ms, TE_eff_ = 30 ms, NSA = 2–5, turbo factor Tf = 4, pixel size: ~150·200 μm^2^, slice thickness = 700 μm) prior to a 3D volume acquisition (TR = 3000 ms, TE_eff_ = 35–82 ms, NSA = 1–2, Tf = 4–14). In the 3D acquisition, the matrix size was adjusted to achieve 160^3^ μm^3^ voxels with minimal field-of-view (FOV) but full tumour coverage. The 3D sequence was repeated twice using voxel sizes of 200^3^ μm^3^ and 240^3^ μm^3^ but with maintained FOV. The methods are henceforth designated 3D-160, 3D-200 and 3D-240, respectively. Saturation slices were always used in the phase encoding direction.

During the MRI experiments anaesthesia was maintained using a mixture of air and isoflurane (1.5–2.0 %) (Isoba vet., Schering-Plough Animal-health, Farum, Denmark). Body temperature was maintained with a heating pad on the animal bed and a pressure sensitive pad was used for respiratory triggering.

### Image processing and tumour mass estimation

**Figure 1:**
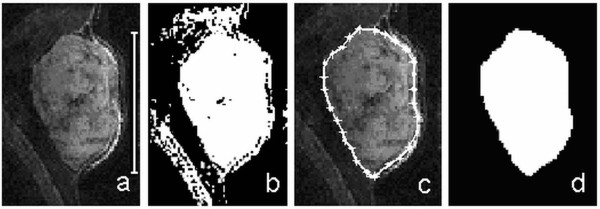
**a) 3D-160 MR image of a subcutaneous GOT1 tumour positioned in the neck of the mouse.** The tumour is visible as the hyper-intense region central to the image, marked by the 1 cm vertical bar. **b)** The global segmentation threshold is applied and regions of failed segmentation appear outside the tumour. **c)** The manual delineation excludes the areas of failed segmentation, and the final result is shown in **d)** where white pixels represent the region that will be accounted for as tumour volume.

The image series were processed on a PC workstation using an in house developed volume estimation tool implemented in MATLAB (R2008b, The MathWorks, USA). Figure 1 shows the volume determination process. The average signal from a manually outlined region containing approximately equal amounts of tumour and background tissue was used for threshold segmentation (Figure 1b). Successful segmentation was ensured by allowing threshold adjustment on an image by image basis, and a polygon function could be used to manually exclude regions where the segmentation had failed (Figure 1b, c). With the tumour border properly defined, regions of low intra-tumour signal intensity, erroneously assigned to the background compartment, were converted using a growing seed algorithm, and the resulting image was used for volume calculation (Figure 1b, d). The number of tumour voxels, multiplied by the voxel volume, was converted to mass assuming a tumour density of 1.0 g/cm^3^. The estimated masses from the 2D and 3D images, denoted *m*_*2D*_ and *m*_*3D*_ respectively, were compared to *m*_*T*_ using regression analysis.

The operator determining the masses from the repeated scans with increasing voxel sizes (*m*_*3D-160*_, *m*_*3D-200*_, and *m*_*3D-240*_) was blinded to images with higher resolution when such existed.

### Intra- and interobserver variability

Repeated (n = 10) segmentation and mass calculation was performed to investigate the intraobserver variability. Five image series including a small tumour (*m*_*T*_ = 0.01, 2D and 3D-160), a medium sized tumour (*m*_*T*_ = 0.10 g, 3D-160) and a large tumour (*m*_*T*_ = 0.87 g, 2D and 3D-160) were included in the assessment. The operator was blinded to the 3D images when evaluating the 2D images. Learning effects were minimized by presenting the image series in a random order and allowing at least several hours to pass between sessions. The coefficient of variation (CV) was calculated for each image series.

Two additional observers repeated the process on the five image series to investigate interobserver variations. Their results were compared to the corresponding average values obtained by observer 1 in the intraobserver assessment.

### Estimation of influence of partial volume effect

A simulation was set up using MATLAB to quantify the maximum influence that PVE could have on volume estimations based on 3D MRI images of a spherically shaped tumour. Tumour diameter and voxel dimensions were used as input parameters. A 3D grid was used to simulate the image matrix, with grid elements acting as image voxels. A sphere, simulating the tumour, was superimposed on the grid with coinciding origins. Grid elements located inside and outside the sphere were defined as tumour and background elements, respectively. Elements intersected by the rim of the sphere were defined as PVE elements. Each one-element thick section of the grid (representing the image slices) was considered for two extreme cases of segmentation, where PVE elements were classified either as tumour elements, or as background elements, thus overestimating or underestimating the true tumour volume, respectively.

The total influence of PVE on the tumour volume estimation was defined as $\frac{\tilde{V}-V}{V}\cdot 100$ %, where $\tilde{V}$ represents the total volume of tumour elements in the simulation and *V* is the true tumour volume. Voxel sizes of 100^3^–250^3^ μm^3^ and 3–9 mm (diameter) tumours were simulated, and the resulting data were presented as relative volume errors for different number of voxels-per-tumour-diameter ratios.

### Statistics

Regression analyses were performed using the Microsoft Excel (2003) statistics toolkit. Pearson’s r squared correlation coefficient was used.

## Results

### Determination of tumour mass

All tumours found at necropsy were well visualized in the MR images. The mass of the resected tumours, *m*_*T*_, was between 0.01 g and 2.28 g. Tumours with *m*_*T*_ <0.2 g are henceforth classified as small.

**Figure 2:**
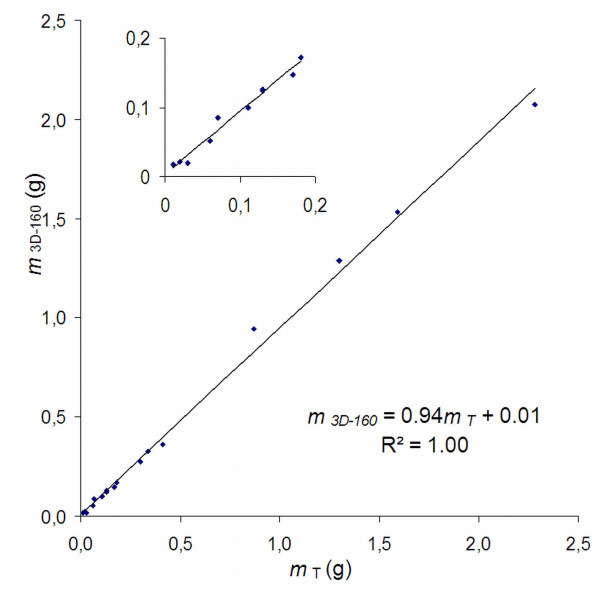
**Tumour mass calculated from 3D-160 MR images,*m***_***3D-160***_**, vs. tumour mass measured after resection,*m***_***T***_**, n = 17.** There is a strong correlation between the parameters. The inserted figure shows the same data when only small tumours (<0.2 g) are included (*m*_*3D-160*_ = 0.90*m*_*T*_ + 0.00, R^2^ = 0.98, n = 10).

A strong correlation was found between the tumour mass estimated from the 3D-160 MR images (*m*_*3D-160*_) and *m*_*T*_ (Figure 2). The correlation was persistent when data only from the small tumours (*m*_*T*_ < 0.2 g) were included.

**Figure 3:**
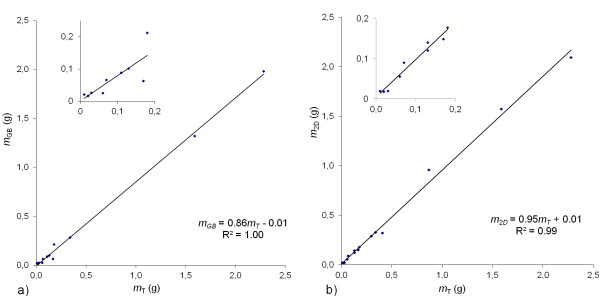
**Tumour mass calculated from a) gauge block measurements,*m***_***GB***_**, and b) 2D images,*m***_***2D***_**, vs. tumour mass measured after resection,*m***_***T***_**. a)***m*_*GB*_ vs. *m*_*T*_ (n = 12). The correlation was strong when all tumour sizes were included (R^2^ = 1.0), but was lower in the assessment of small tumours only (inserted figure; *m*_*T*_ <0.2 g, n = 9, *m*_*GB*_ = 0.78*m*_*T*_ + 0.00, R^2^ = 0.65). The corresponding correlation for *m*_*3D-160*_ vs. *m*_*T*_ for the same set of tumours was *m*_*3D-160*_ = 0.93*m*_*T*_ + 0.00, R^2^ = 1.00 (*m*_*T*_ <0.2 g: *m*_*3D-160*_ = 0.90*m*_*T*_ + 0.00, R^2^ = 0.97). **b**) *m*_*2D*_ vs. *m*_*T*_ (n = 15). The correlation was strong when all tumour sizes were included (R^2^ = 0.99), and persisted in the assessment of small tumours only (inserted figure, *m*_*T*_ <0.2 g, n = 9, *m*_*2D*_ = 0.94*m*_*T*_ + 0.00, R^2^ = 0.96). The corresponding correlation for *m*_*3D-160*_ vs. *m*_*T*_ for the same set of tumours was *m*_*3D-160*_ = 0.93*m*_*T*_ + 0.01, R^2^ = 1.0 (<0.2 g: *m*_*3D-160*_ = 0.91*m*_*T*_ + 0.00, R^2^ = 0.98).

In twelve mice, tumours were measured with gauge blocks in addition to MRI and digital balance. The correlation between *m*_*GB*_ and *m*_*T*_ was strong when data from all tumours were included (Figure 3a). When analysing small tumours only, the correlation was markedly reduced (R^2^ = 0.65). The same subset of tumours analysed with the 3D-160 method yielded R^2^ = 0.97.

Comparing *m*_*2D*_ to *m*_*T*_ (n = 15, one image series was lost, and one did not cover the entire tumour) a strong correlation was obtained (R^2^ = 0.99) (Figure 3b). Also for small tumours a strong correlation was found (R^2^ = 0.96), i.e. the 2D method was comparable to the 3D-160 method regarding accuracy.

To exclude the inherent uncertainty of the digital balance, *m*_*2D*_ was compared directly to *m*_*3D-160*_. The linear relation in the regression and the correlation approached unity *m*_*2D*_ = 1.01*m*_*3D-160*_–0.00, R^2^ = 1.00, n = 15. For small tumours the corresponding relation was *m*_*2D*_ =1.04 *m*_*3D-160*_ + 0.00, R^2^ = 0.99, n = 9.

The analysis of *m*_*3D-160*_, *m*_*3D-200*_, and *m*_*3D-240*_ vs. *m*_*T*_ did not reveal any obvious voxel size dependence in the range of tumour- and voxel sizes investigated. The acquisition time required for the smallest tumour studied (*m*_*T*_ = 0.01 g) was clearly reduced with larger voxels: 55, 34 and 22 minutes for the 160^3^, 200^3^ and 240^3^ μm^3^ images, respectively.

### Intra- and interobserver variability

The intraobserver variation was in general low for each method and tumour size (CV of 2–3 %), but an indication of increased variability (CV of 7 %) was observed in the 2D image set of the smallest tumour (Table 1).

**Table 1:** The coefficient of variation (CV) calculated for the five intraobserver variability assessments. The CV is based on 10 volume calculations performed on each of the five image series

***m***_***T***_**(g)**	**Method**	**CV (%)**
0.01	3D-160	3.1
	2D	6.9
0.10	3D-160	3.1
0.87	3D-160	2.1
	2D	1.9

The interobserver results indicated a difference between the 2D and 3D-160 methods (Table 2). The relative deviation from the average mass was higher when determined from the 2D images compared to the 3D-160 images. The effect was more pronounced for the smallest tumour size.

**Table 2:** Interobserver variation. The interobserver variation of tumour mass measurements for three observers given as relative deviation (in per cent) from the average value of the mass (n = 10) obtained by observer 1 for three different tumours and two imaging methods studied

**Mean*m*(g)**	**Method**	**Interobserver variation (%)**		
		**Obs1**	**Obs2**	**Obs3**
0.02	3D-160	−	0.7	−4.7
0.02	2D	−	23	11
0.10	3D-160	−	1.2	−4.3
0.91	3D-160	−	−1.4	0.1
0.99	2D	−	2.2	−9.0

### Computer simulation

Table 3 shows data from the simulations of the maximum possible influence that PVE could have on volume estimation. The largest relative volume error was approximately 40 % for a voxels-per-tumour-diameter ratio of 12, corresponding to, e.g., a tumour of 3 mm diameter (~0.01 g) imaged with the voxel size set to 250^3^ μm^3^, i.e. the smallest tumour included in this study, in combination with the largest voxel size used in the imaging experiments.

**Table 3:** Influence of partial volume effect on volume determination. The maximum possible influence of the partial volume effect (PVE) on tumour volume estimations, assuming isotropic voxels and a spherical tumour, determined by simulations

**Voxels/diameter(mm**^**−1**^**)**	**Maximum relative volume difference (%)**	
	**overestimated**	**underestimated**
70	6	−6
45	10	−10
28	16	−14
12	39	−31

The voxels-per-tumour-diameter ratio is listed with the corresponding results from the simulation. The relative difference between over- and underestimated tumour volumes and the analytically calculated volume are given as a percentage of true tumour volume.

## Discussion

The present study evaluated the possibilities and requirements for accurate measurement of small tumour sizes in mice using MRI. A linear, highly correlated relationship between weighed and MRI measured tumour masses down to 10^−2^ g was found. 3D acquisition should be considered when tumour masses of 10^−1^ gram or less are expected, due to the relative increase of PVE. In very small tumours (10^−2^ g) image acquisition at high resolution (in our setup 160^3^ μm^3^ voxels) should also be considered. The increased acquisition time using high resolution is compensated for by the smaller FOV needed to cover the tumour. Short acquisition times allow either additional MR investigations on the same animal, such as determining tumour diffusion, perfusion or metabolic parameters within one MR session, or a higher animal throughput.

Initially, the 2D method was included only for anatomical reference since gradient performance limited the minimum slice thickness to 700 μm, resulting in substantial PVE. However, the accuracy of volume estimations based on the 2D images was similar to that based on the 3D images (Figures 2 and 3b). This might be due to the possibility to study adjacent image slices, which probably improves the view of the tumour shape and delineation of the tumour border. However, the 2D method has a higher interobserver variability (Table 2), which supports the use of a 3D method for very small tumours, in order to limit the subjectivity in the evaluation.

In the 3D method, the turbo factor (Tf) was adjusted to reduce acquisition time. An increase in Tf results in an increased point spread function (PSF). Computer simulations assuming similar acquisition parameters as those used, and T2 values common at 7 T [23] showed that the PSF was broadened only by a factor of 1.6 compared to the value for Tf = 1 (data not shown). The minimum TE_eff_ is also affected by the Tf, i.e. the image contrast will vary slightly with Tf. However, tumours were always easily visualized and, altogether, the range of Tfs used in the study might only affect the results to a minor extent.

The most time consuming process in the volume determination was probably when adjusting the threshold value in images where the global segmentation had failed. To reduce the time of analysis one could e.g. calculate an average volume based on two extreme segmentations; one including most of the border, and one excluding it. Such a procedure would, however, overestimate the volume, especially for small voxel-to-diameter ratios, where asymmetry between over- and underestimated volume errors is more pronounced (Table 3). In situations when small tumours require polygon delineation of the tumour border, the decision to assign voxels intersected by the polygon line to the tumour or the background compartment, will require asymmetry consideration since it might have a significant effect on the volume estimation (Table 3).

The tumour density assumption (1.0 g/cm^3^) might be an underestimation that would account for the fact that the relations between the determined masses (*m*_*3D-160*_*, m*_*2D*_ and *m*_*GB*_) and the weight (*m*_*T*_) were less than unity. Another contributing factor could be the inherent uncertainty in the digital balance, since errors in the predictor used in regression analysis are typically manifested as a decrease of the slope coefficient towards zero [24]. These two considerations are justified by the fact that the slope was 1.01 when *m*_*3D-160*_ and *m*_*2D*_ were compared directly to each other, thus excluding the density effect and predictor uncertainties.

Few studies were found in the literature where the accuracy of MRI based tumour size measurement was verified by e.g. comparison with weight after resection. He et al. found a correlation of R^2^ = 0.96 (n = 7) when comparing volumes of pancreatic tumours in mice from T2-weighted 2D MR images (similar to our 2D method), acquired at 4.7 T, for 0.2–2.0 g tumours [6]. We obtained a similar correlation (R^2^ = 0.96, n = 9) but for smaller tumours (0.01–0.2 g) since we used six times smaller voxel volume, i.e. the influence of PVE might be comparable. Other groups have reported MRI tumour size measurements in mice but without verification with tumour weight, e.g. [7-9]. One group reported MRI measurements of tumours in mouse pancreas down to 0.14 g at 7.0 T using sequence parameters similar to our 2D method (T2-weighted RARE sequence, 0.015 mm^3^ voxels), but verified the volume determination by one phantom measurement only [9].

Using high resolution microCT (7 min acquisition time, voxel size of 81^3^ μm^3^, voxel volume of 0.0005 mm^3^) a close correlation was found for 0.02–0.25 g s.c. tumours in mice, verified by weight after resection (R^2^ = 0.97, n = 20) [12]. Thus, microCT is a fast and accurate method, but the absorbed dose delivered to the animal, and especially to the tumour tissue, is a confounding factor in therapy response assessments.

The generally faster T1-weighted sequences have also been used for tumour imaging (e.g. [8]). Often however, the tumour and surrounding tissue have similar T1-values, thus requiring use of contrast agents. T1-weighted sequences without contrast agents may be useful for imaging tumours in e.g. the bladder wall, where fluid constitutes the surrounding tissue, due to widely different T1-values in fluid and solid tissue [7].

## Conclusion

This study shows the feasibility of accurate and precise MRI based size measurement of small tumours in mice. Furthermore, short acquisition times that allow additional MR investigations within the same session can be realized by careful selection of MR sequence parameters, e.g. using 2D instead of 3D methods and optimal voxel size.

## Competing interests

The authors declare that they have no competing interests.

## Authors’ contributions

MM designed and carried out the imaging studies, performed the statistical analysis and drafted the manuscript. ML participated in designing and carrying out the imaging study, approved the statistical analysis and helped to draft the manuscript. MH conceived of the study, and participated in its design and coordination. EFA participated in designing the study, performing the statistical analysis and helped to draft the manuscript in addition to supervising the study coordination. All authors read and approved the final manuscript.

## Pre-publication history

The pre-publication history for this paper can be accessed here:

http://www.biomedcentral.com/1471-2342/12/12/prepub
